# Supplementary figures and images for: TEM8/ANTXR1-specific CAR T cells mediate toxicity in vivo
Source: PLoS One. 2019 Oct 17;14(10):e0224015. doi: 10.1371/journal.pone.0224015 (PMC6797195; doi:10.1371/journal.pone.0224015)

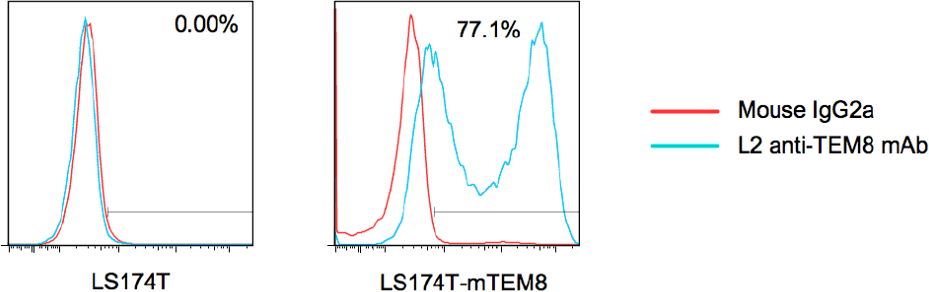

Supplement: S1 Fig — LS174T cells transduced with a lentivirus expressing the murine form of TEM8 were stained with the TEM8 specific L2 monoclonal antibody (80ug/ml) or a concentration- and isotype-matched antibody control (clone ZX4, Thermo Fisher Scientific). They were then stained with a phycoerythrin-conjugated goat anti-mouse IgG antibody (Biorad) and analysed by flow cytometry using an LSRII Cytometer (Becton Dickinson) and FlowJo software (Tree Star). (TIF) [file pone.0224015.s001.tif]

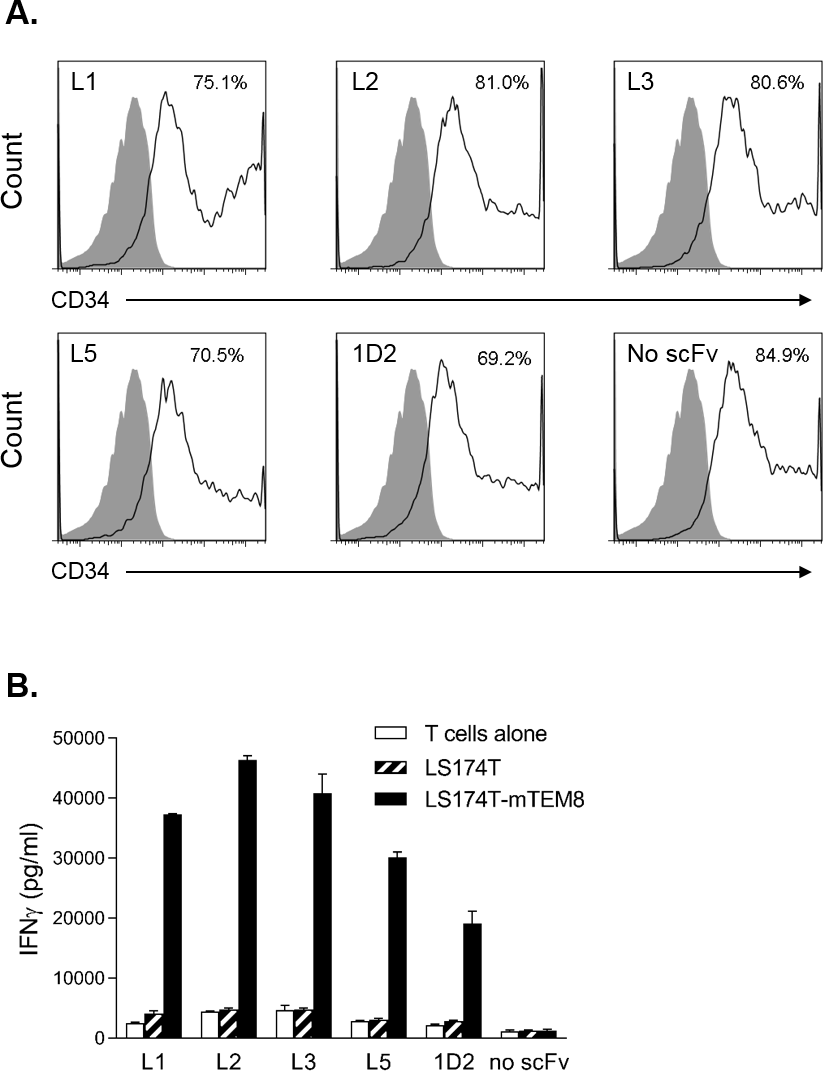

Supplement: S2 Fig — (A) Expression of all 5 TEM8-specific CARs (L1, L2, L3, L5 and 1D2) and the no scFv control CAR in mouse T cells was demonstrated by flow cytometry staining for the coexpressed CD34 marker. % values show proportion of cells stained for CD34 in transduced T cells (black line) compared to mock-transduced T cells (shaded). (B) Antigen specific responses to LS174T cells expressing mouse TEM8 were detected using a mouse IFN-gamma ELISA platinum kit (Invitrogen). CAR-T cell lines were diluted with mock-transduced T cells to equalise for transduction efficiency. The graph shows the mean of duplicate cultures (± standard deviation, SD). (TIF) [file pone.0224015.s002.tif]

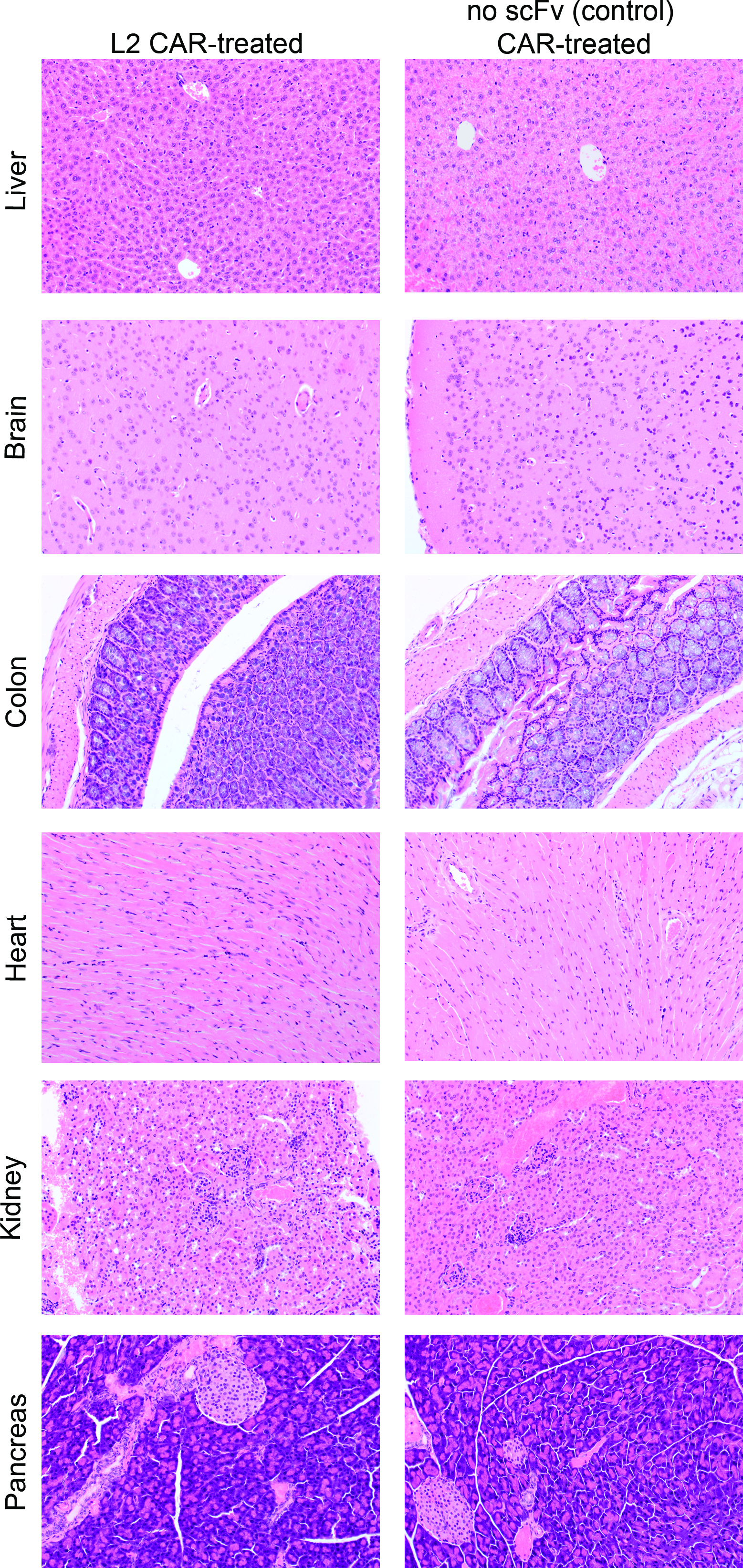

Supplement: S3 Fig — Mice (n = 3 per group) were injected with an effective dose of 11.1 million or 12.6 million T cells that all expressed the L2 or no scFv CAR respectively. Tissues were taken 3 days later. (Magnification = x200). (TIF) [file pone.0224015.s003.tif]
